# Factors associated with resilience among children and youths with disability during the COVID-19 pandemic

**DOI:** 10.1371/journal.pone.0271229

**Published:** 2022-07-29

**Authors:** Afiqah Yusuf, Nicola Wright, Mandy Steiman, Miriam Gonzalez, Arun Karpur, Andy Shih, Keiko Shikako, Mayada Elsabbagh

**Affiliations:** 1 Azrieli Centre for Autism Research, Montreal Neurological Institute-Hospital, Montreal, Quebec, Canada; 2 Biostatistics and Health Informatics, Kings College London, London, United Kingdom; 3 Autism Speaks, New York, New York, United States of America; 4 School of Physical and Occupational Therapy, McGill University, Montreal, Quebec, Canada; Radboud University: Radboud Universiteit, NETHERLANDS

## Abstract

There is evidence of negative impact of social distancing and confinement measures to manage the COVID-19 pandemic on children, including increased anxiety and depression and behaviour difficulties. Paradoxically, positive impacts like increased support and more self-care activities have also been documented. Little is known about the impact of the COVID-19 pandemic on the children with disability and the potential role of familial, environmental, and biological factors on mitigating this impact. The aims of the study were 1) identifying profiles of functioning across multiple domains during the COVID-19 pandemic and 2) examining the extent to which parenting self-efficacy, support in accessing schooling, and type of diagnosis predict the likelihood of resilience among children with disability, after controlling for household income and single-parent status. An online survey developed from COVID-19 guidance recommendations, was available from June 11- July 21, 2020, and resulted in a convenience sample of caregivers across Canada (*n* = 883) of children with disability (mean age of 9.4 years old, SD_age_ = 5.7, 58% male). We conducted latent class analysis to examine the number of latent profiles on caregiver-reported changes of 12 functioning domains, as either ‘worsening’, ‘no change’, or ‘improving’. Most participants belonged to ‘stable’ or ‘worsening’ profiles. However, we identified a small subgroup with improvements in child functioning, a pattern indicative of a ‘resilient’ profile. Using a multinomial logistic regression, we found that diagnosis type, parenting self-efficacy and support in accessing schooling were associated with membership in the Resilient or Stable profiles compared to the Worsening profile, after controlling for single-parent status and income. Taken together, our findings identified variability in responses to adversity that is dependent on the child’s diagnosis type, parenting self-efficacy, and support in accessing schooling. By identifying potentially modifiable predictors of resilience, namely parenting self-efficacy and support in accessing schooling, we signal the potential for tailored supports for different diagnoses, through interventions that enhance caregiver empowerment, access to schooling, access to health and social services, and/or mitigate disparities resulting from social disadvantage.

## Introduction

Following the declaration of the COVID-19 pandemic by the World Health Organization in March 2020 [1], several countries imposed intensive public health measures to manage the outbreak. These public health measures consisted of enforced physical distancing and increased home confinement through the closure of school and daycare, some health and social services, non-essential businesses, workplaces, and gathering places, and in some cases, imposed curfews. Restrictive public health measures were gradually lifted or imposed over time depending on the severity of outbreaks, evolving into a hybrid of virtual and in-person modes of delivery of education, health, and social services for children and youths. While there were concerns of a negative impact on children’s and youths’ mental health and wellbeing stemming from the pandemic, including increased anxiety, depression [2], and behaviour difficulties [3], a systematic review and meta-analysis of 65 longitudinal studies suggest that most mental health symptoms increased early in the COVID-19 pandemic (March and April 2020) but general mental health and anxiety stabilized to pre-pandemic levels by May to July 2020 across all age groups [4].

Evidence of the impact of the COVID-19 pandemic among children and youths with disabilities is scant. In the broader disaster literature, children with disabilities have a higher risk of exposure, are less likely to receive needed services following disasters or conflict, and are less likely to be considered in emergency plans [5]. Any potentially detrimental impact of the COVID-19 pandemic may also be amplified among children with disability due to greater healthcare needs, dependency on community-based services, and pre-existing mental health concerns [6]. The disruptions in intervention delivery, limited access to families’ social support networks, closure of schools, daycares and/or health and social services due to the pandemic are expected to exacerbate pre-existing challenges for children with disability [7]. The few studies available suggest increased vulnerability to the COVID-19 pandemic among children and youths with disability in the presence of other risk factors, including psychosocial problems and impaired functioning [8]. Other factors that increase vulnerability among children and youths with disability include caregiver depression, anxiety, and stress [9], which are elevated in parents of children with disabilities compared to parents of children without disabilities [8].

At the same time, some studies have not found a differential impact of the COVID-19 pandemic among some children and youths with disability [10–14]. One longitudinal study did not find significant differences compared to pre-pandemic levels in caregiver distress, caregiver life satisfaction, nor internalising or externalising problems in children with intellectual disability [15]. When asked about “silver linings” from the pandemic, some parents of children with intellectual and developmental disabilities reported having more time together as a family, enjoying “a slower pace of life,” being more able to implement behavioural strategies, routines and schedules and engage in enjoyable fun activities and noticing that their child continued to make developmental gains [16]. Therefore, findings to date may be consistent for distinct profiles who respond differently to the COVID-19 pandemic.

These previous studies indicate that a subgroup of children and youths with disabilities may experience “better-than-expected” outcomes in the face of adversity, a pattern previously described as *resilience* [17, 18]. Resilience is not a static trait, rather it is a dynamic process inferred from an individual’s trajectory of functioning following traumatic stressors [18]. Resilience is associated with outcomes reflecting low levels of psychopathology in an individual, such as the absence of symptoms of mood disorders and post-traumatic stress disorder, following a traumatic event [19]. Resilience can also result in positive academic or social achievements [20]. Depending on the domain of functioning measured, certain individuals may show maintenance or even improvement in functioning, whereas others show worsening in functioning following adversity. For example, Bal et al. (2021) found 36% of autistic adults meeting their criterion for “resilience” when defined as individuals who reported “minimal-to-mild” distress despite experiencing “moderate-to-severe” negative impact on two or more domains in their lives [21]. In contrast, when defined as improvements in particular domains of functioning, the prevalence of resilience falls to 3–15% among autistic children and adolescents, depending on the domain [13], although we cannot discount that the difference in age of the sample between studies can also account for this variation.

Thus, characterizing the variability in responses to adversity over a range of domains of functioning using a data-driven approach would provide a more accurate and richer picture of the response to the pandemic. Describing the variability in responses to adversity would also inform the understanding of resilience in this population of children with disabilities. Specifically, identifying the characteristics and contexts that differentiate a “resilient” subgroup can potentially inform modifiable targets for intervention to promote resilience in children. Factors that predict resilience in children with disabilities are not yet well understood. We considered potential factors that confer resilience in children across multiple levels, namely familial, biological, or environmental [22].

Parenting self-efficacy, defined as a parent’s “belief in their ability to perform the parenting role successfully” [23], is one *familial* predictor of resilience that has been linked to use of more optimal parenting and positive behavioural outcomes in both typically developing children and children with disabilities [24, 25]. In the context of the COVID-19 pandemic, parents’ own coping and their ability to help their child to cope may be particularly salient and therefore be an important familial factor in conferring resilience in the child.

There is also evidence that children and youths with specific neurodevelopmental conditions such as autism spectrum disorder (ASD) and intellectual disability (ID) may experience vulnerability during the pandemic [26] including reporting greater problem behaviours [27, 28], worsening sleep [29], increased difficulty in daily activities [10] during the COVID-19 pandemic compared to before the pandemic, suggesting a *biological* predictor of resilience. At the same time, these conditions, namely ASD are heterogenous, with profiles of unique strengths among individuals that may bolster positive responses to the COVID-19 pandemic for some [30]. Taken together, these studies demonstrate that children and youths with ASD and ID are potentially differentially impacted by the COVID-19 pandemic; however, a study comparing them to children with other disabilities is needed.

Other factors thought to be linked with resilience are *external*, including access to environmental resources that can offset the detrimental impact of an adversity [31]. One of the most consistent environmental factors in fostering resilience in children in the context of other adversities (i.e. disaster, war, terrorism) is the availability of a safe environment for children to play and learn including schools and child-care facilities, because these institutions can help reinstitute structure and routines, offer caregiver respite, and afford opportunities for social interactions [32]. The swift resumption of schooling was the endorsed as one of the best post-disaster practices by humanitarian agencies [33]. It is also possible that support offered in settings outside the home, such as a school or community centre, provides additional coping pathways that may promote resilience. One study reported that the absence of indirect school support during the pandemic tended to be associated with an increased likelihood of autistic children exhibiting more behaviour problems [10]. Following the destruction of schools due to an earthquake in L’Aquila, Italy, parents reported loss of social, educational, and behavioural skills in their autistic children, and this decline was confirmed on their adaptive functioning scores [34]. No other studies have examined the role of access to schooling, in promoting resilience among children with neurodevelopmental conditions during the COVID-19 pandemic.

Recent findings have highlighted the disproportionate impact of the COVID-19 pandemic on marginalized populations [35]. Income level has a cascading effect on pandemic impact, with families who have low incomes being at increased risk of exposure to outbreaks [36], more likely to experience job loss during the pandemic [37], and experience worsened impact of the pandemic on their mental health [38]. Single parents are also more likely to report greater exhaustion during the pandemic [39]. Overall, this increased burden on an already disadvantaged population may explain the association between low-income families and single-parent households showing greater risk of psychosocial problems in their child during the COVID-19 pandemic [8].

In the current study we investigated resilience in the context of the COVID-19 pandemic focusing on children with disabilities by using a data-driven approach to characterize profiles of functioning across multiple domains. We also examined the potential impact of familial, environmental, and biological factors on these response profile. Specifically, the aims of the study were 1) to identify subgroups of children who show different profiles of functioning across multiple domains during the COVID-19 pandemic and 2) to examine the extent to which parenting self-efficacy, ease in accessing schooling, and presence of a specific neurodevelopmental condition (ASD or ID) predict the likelihood of resilience among children with disabilities, after controlling for socio-demographic factors (household income and single-parent status). Based on the studies cited above, we hypothesized that higher parenting self-efficacy, greater ease in access to schooling, and the absence of neurodevelopmental conditions are associated with a greater likelihood to show resilience, after controlling for income and single-parent status.

## Methods

### Study design and participants

We used data collected in Canada for the Global Report on Developmental Delays, Disorders and Disabilities. The Global Report is an ongoing initiative led by the World Health Organization, UNICEF, and Autism Speaks to document the experiences of caregivers of children with developmental delays, disorders, and disabilities globally. Following the COVID-19 pandemic, topics such as response to the pandemic and receipt of pandemic-related services and supports were added using survey questions based on COVID-19 policy guidance recommendations for persons with disabilities [40–43]. In Canada, the survey was developed, piloted, and disseminated in collaboration with caregivers of children with disabilities and was available in both English and French.

We used a cross-sectional design and disseminated the Global Report survey using an online crowdsourcing sampling strategy through networks of researchers and parents. The survey was active from June to July 2020 and collected information from a non-random, convenience sample of caregivers of children with developmental delays, disorders, and disabilities from across Canadian provinces and territories. Caregivers were offered $15 in appreciation for their participation.

### Ethics approval

The Research Ethics Office (Institutional Review Board) of the Faculty of Medicine and Health Sciences at McGill University approved this study (study ID: A10-M75-12B). Written informed consent was obtained for every participant.

### Variables selected for the present study

The specific survey items that are the focus of the current paper are detailed as follows.

Parenting self-efficacy in the context of the COVID-19 pandemic was measured using the question “In general, how confident are you that you can help your child with a disability cope with during the pandemic?”. The response option to this question is a five-point scale from “Not at all confident” to “Extremely confident”. In line with recommendations on measuring specific forms of self-efficacy, a measure of high specificity is most relevant because we aimed to explain a particular level of child functioning in the given context of the COVID-19 pandemic.

Presence of ASD or ID versus other conditions was assessed by diagnoses reported for the child in the household with greater needs. The participant was asked if the child with the greater needs has been diagnosed with any of the following conditions: autism spectrum disorder, intellectual disability, anxiety, depression, epilepsy, seizures, allergies, vision/hearing problems, troubles with mobility, chronic breathing difficulties, gastrointestinal difficulties, eating disorder, sleep disorders, and other. Diagnoses of autism spectrum disorder or intellectual disability were categorized as neurodevelopmental conditions of interest.

Ease in accessing schooling was measured using the item “Getting daycare, preschool or school for your child” under the question stem “During the pandemic, how easy has it been to maintain help and support for your family?”. A five-point response option to this item ranged from “Very easy” to “Very difficult”.

Sociodemographic variables of interest were two-parent status and household income. While other socioeconomic factors such as caregiver education, age, and gender could play a role in children’s resilience, our focus on two-parent status and household income lies in the disproportionate impact of the COVID-19 pandemic on populations with low income and single-parent households.

The outcome variable, child functioning was assessed using the question stem, “During the pandemic, did you notice changes in your child in any of the following areas?” Caregivers were to rate either “Worsened,” “Stable,” or “Improved” on each of the 12 domains of child functioning listed below:

1. Troubling behaviours (e.g., self-injury, aggression, tantrums)
2. Daily living skills (e.g., using the bathroom, dressing themselves, feeding themselves)
3. Health problems (e.g., seizures, illness)
4. Mental health problems (e.g., anxiety)
5. Sleep problems (e.g., trouble falling asleep or staying asleep)
6. Diet/eating difficulties
7. Social interaction difficulties (e.g., responding to name, making friends)
8. Repetitive behaviours/limited interests/insistence on sameness
9. Communication difficulties (e.g., language delay, cannot explain their emotions)
10. Safety concerns (e.g., getting in trouble with police, neighbors, strangers)
11. Sensory issues (sensitivity to certain sounds or lights)
12. Education (e.g., academic achievement).

### Statistical analysis

Descriptive statistics were run to examine group-level statistics on respondent sociodemographic and on variables of interest.

Latent class analysis (LCA) was used to create groups of children who showed different profiles of functioning in the pandemic. LCA, a type of mixture modelling, assumes that an underlying categorical variable explains the relationships among item responses, and uses response patterns in the data to identify profiles, or latent classes. The approach is “person-centred” using patterns of response to create groups of individuals, as opposed to factor analysis which is “variable-centred” and groups items together. A series of LCAs were run to examine the underlying number of latent groups on the 12 functioning questions using Mplus version 8 [44]. A series of models with different numbers of classes (e.g. two-class, three-class) were estimated and the absolute and relative fit indices of these models were compared. The Akaike’s Information Criteria (AIC), Bayesian information criterion (BIC), the Sample-adjusted Bayesian Information Criterion (SBIC) and the Vuong-Lo-Mendel-Rubin (LMR) test statistics were used as statistical criteria to compare models to identify the optimal number of groups to retain [45]. Lower AIC, BIC and SBIC values in model-to-model comparisons reflected a better fit. A nonsignificant chi-square value (*p* < .05) for the LMR statistic suggests that a model with one fewer class is preferred. Further, average posterior probabilities and entropy values equal to or greater than .80 indicate clear classification and greater power to predict class membership [46].

We then extracted the class membership groupings and used this as the dependant variable in a multimonial logistic regression in SPSS version 25 [47] to examine associations between class membership and the hypothesised relevant variables. Multinomial regression is an extension of binomial logistic regression which allows for more categories. Separate regression models were run for parenting self-efficacy, type of diagnosis and ease in access to schooling. All models controlled for two-parent status and income level.

#### Data validation

Upon survey closure, the dataset (n = 2,133) was verified for invalid responses using a two-stage screening process. In the first stage, we identified potentially invalid cases by checking for: (1) duplicate IP addresses, (2) incorrect responses in free text fields (e.g., respondent’s name), (3) duplicate responses in open-ended questions, (4) completing the survey in less than 10 minutes, (5) impossible time gap between respondent and child age, and (6) cases where the same answer (e.g, “b”) was selected repeatedly.

In the second screening stage, potentially invalid cases were removed when the following criteria were satisfied: (1) inconsistency between name in consent, parental gender, and relationship to child, (2) inconsistent responses to questions related to child age at diagnosis (e.g., age when concerns were first noticed, age at diagnosis), (3) a period or no space in the name provided for consent purposes, (4) an email address that started with the full name and was followed by numbers, (5) a date of birth in the consent date field, and (6) postal codes reported in U.S.A format.

All cases that were considered invalid based on the screening criteria were deleted from the dataset, resulting in a total of 883 valid responses.

## Results

### Descriptives

Table 1 describes the demographics of the sample who responded to the survey. Most respondents were a mother to a person with disability, were in a two-parent household, were White, and reported an annual household income of more than or equal to $40,000 CAD. The respondent’s child with disability was on average 9.4 years old (*SD* = 5.7) and was approximately evenly split between males and females. More than half of the respondents reported a diagnosis of ASD or Intellectual Disability (ID) in their child. Thirty-seven percent of respondents reported at least one other diagnosis in addition to the ASD/ID diagnosis. The most common co-occurring diagnoses in the children/youths with ASD/ID were anxiety (21.5%), sleep disorders (17.9%), troubles with mobility (14.9%) and epilepsy (13%). The remaining 44% of respondents reported no ASD/ID but other conditions, with the most reported diagnoses as follows: troubles with mobility (10.8%), anxiety (9.6%), epilepsy (9.5%), and vision/hearing problems (8.8%).

**Table 1:** Demographics of the survey respondents (*n* = 883).

Sociodemographics	n	%
Relationship to person with disability		
Biological mother	533	61.3
Biological father	236	27.2
Other	100	11.5
Household status		
Two-parent household	701	80.9
Single-parent household	158	18.2
Other	7	0.8
Annual household income		
< = $39,999	106	12.3
> = $40,000	755	87.7
Education of respondent		
High school or less	76	8.8
Undergraduate degree or diploma	544	63.1
Higher education or professional degree	216	25.1
Other	25	2.9
Ethnicity of respondent		
Indigenous	214	24.7
White	537	61.9
Asian	46	5.0
Black	28	3.2
Other	42	4.8
Gender of child/youth with disability		
Male	502	57.8
Female	365	42.1
Other	1	0.1
Diagnoses of child/youth		
ASD/ID only	168	19.2
ASD/ID plus other diagnoses	322	36.9
Diagnoses other than ASD/ID e.g., troubles with mobility, anxiety, and epilepsy	383	43.9

### Caregiver-reported change in functioning during the pandemic

No change in functioning in the child or youth was the most frequently endorsed response (50%), followed by worsening (31%) and then improving (19%).

Table 2 details the caregiver-reported change in their child by domain of functioning. Eighty-six percent of caregivers reported a worsening in at least one domain of functioning in their child during the pandemic, with 80% reporting worsening in two or more domains in functioning. Most domains were endorsed as having worsened consistently by 27–36% of the sample. The domain endorsed by the most caregivers as having worsened was sleep problems (38.1%), followed by mental health problems (35.5%), repetitive behaviours/limited interests (33.7%%), and social interaction difficulties (32.0%). The domain endorsed by the fewest caregivers as having worsened was health problems (18.9%), followed by sensory issues (23.3%) and safety concerns (23.9%).

**Table 2:** Frequency and percentage of caregiver-reported changes in their child by domain of functioning.

	Worsened		No change		Improved	
	n	%	n	%	n	%
**Troubling behaviours**	256	29.1	526	59.8	98	11.1
**Daily living skills**	233	26.5	428	48.6	219	24.9
**Health problems**	166	18.9	539	61.4	173	19.7
**Mental health problems**	311	35.5	404	46.1	161	18.4
**Sleep problems**	335	38.1	389	44.2	156	17.7
**Diet/eating difficulties**	232	26.8	475	54.8	160	18.5
**Social interaction difficulties**	281	32.0	437	49.7	161	18.3
**Repetitive behaviours/limited interests/insistence on sameness**	294	33.7	438	50.2	140	16.1
**Communication difficulties**	251	28.5	472	53.6	157	17.8
**Safety concerns**	210	23.9	543	61.8	125	14.2
**Sensory issues**	204	23.3	521	59.4	152	17.3
**Education**	274	31.2	448	51.0	156	17.8

In contrast, sixty-six percent of caregivers reported an improvement in at least one domain of functioning in their child during the pandemic, with 51.9% reporting improvement in two or more domains in functioning. The domain most reported as improved was daily living skills (24.9%), followed by the domains “health problems” (19.7%), “diet/eating difficulties” (18.5%), and “social interaction difficulties” (18.3%). The domain least frequently reported as improved was troubling behaviours (11.1%), followed by safety concerns (14.2%) and repetitive behaviours/limited interests (16.1%).

### Latent class analysis

**Fig 1:**
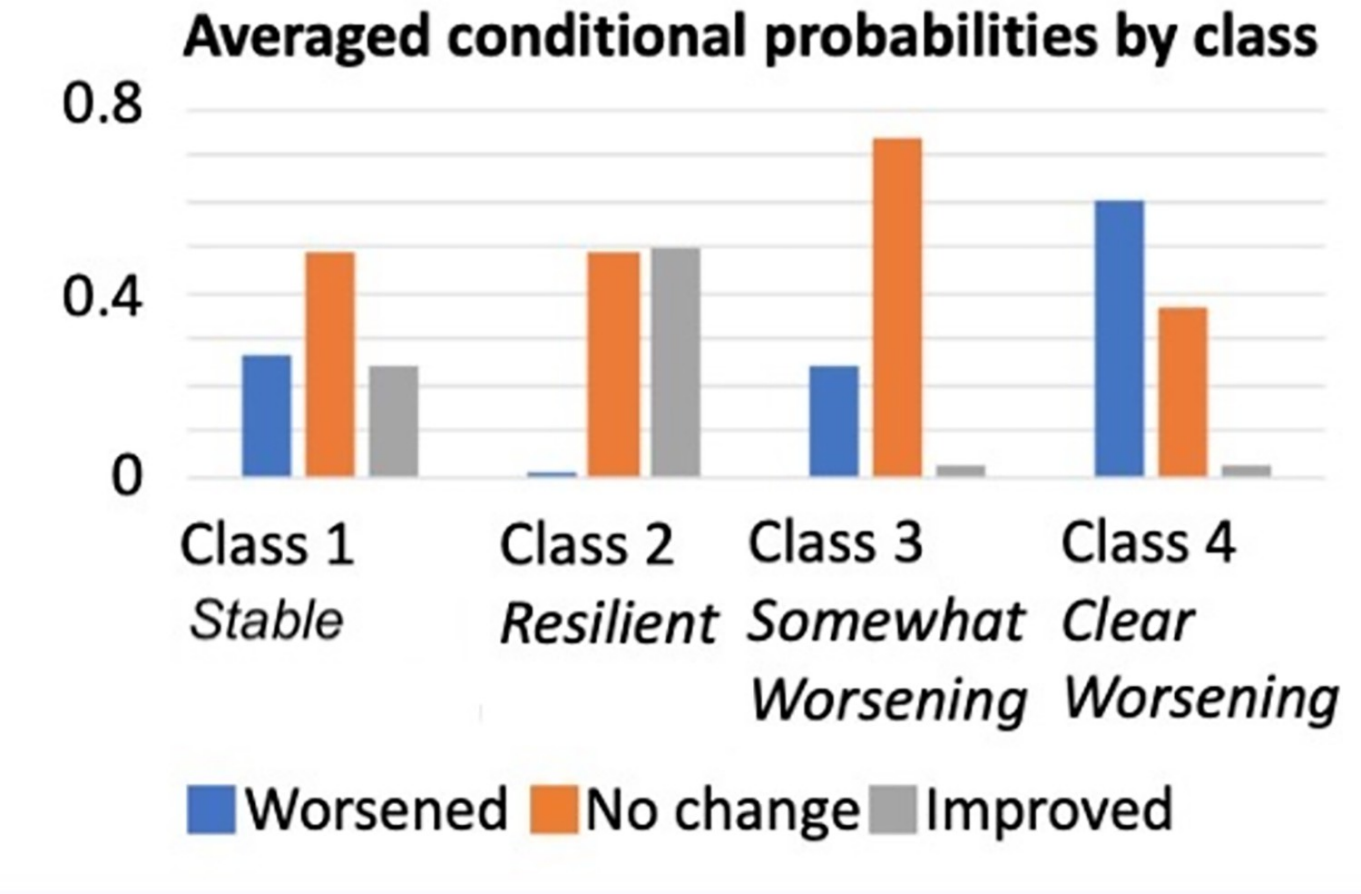
Averaged conditional probabilities by class.

We conducted latent class analysis to examine the underlying number of latent classes on caregiver-reported changes of the 12 functioning domains. The absolute fit statistics decreased from the three-class (BIC = 20,057.23, SBIC = 19822.22, AIC = 19703.35) to the four-class (BIC = 19,987.90; SBIC = 19,673.50; AIC = 19514.47) and increased again for the five class (BIC = 20,029.57, SBIC = 19635.77, AIC = 19436.58). In addition, the V-LMR test statistic fell out of significance for the five-class model (p = .764). Thus, the four-class model better represented the data based on the absolute and V-LMR statistics. The mean posterior probability scores ranged from .87 to .91 and the entropy value was .80. The conditional probabilities for the items for each latent class are shown in Table 3 and the averaged conditional probabilities for each of the four classes are visualized in Fig 1.

**Table 3:** Response profiles (i.e., classes) derived from the latent class analysis with probability of response for each domain.

Domain	Class 1: Stable profile (n = 437)			Class 2: Resilient profile (n = 90)			Class 3: Somewhat worsening profile (n = 237)			Class 4: Clear worsening profile (n = 145)		
	Wor-sened	No change	Improved	Wor-sened	No change	Improved	Wor-sened	No change	Improved	Wor-sened	No change	Improved
**Troubling behaviour**	0.216	0.636	0.147	0.025	0.604	0.371	0.215	0.78	0.004	0.805	0.176	0.019
**Daily living**	0.313	0.371	0.316	0	0.287	0.713	0.154	0.791	0.055	0.461	0.455	0.084
**Health problems**	0.251	0.443	0.306	0	0.564	0.436	0.136	0.851	0.014	0.205	0.773	0.022
**Mental health**	0.271	0.456	0.264	0.03	0.497	0.474	0.335	0.658	0.007	0.837	0.136	0.027
**Sleep**	0.314	0.444	0.242	0.02	0.535	0.446	0.41	0.551	0.038	0.75	0.201	0.049
**Diet/eating difficulties**	0.261	0.523	0.216	0.034	0.237	0.729	0.231	0.738	0.031	0.489	0.496	0.014
**Social interaction difficulties**	0.283	0.43	0.287	0.023	0.531	0.446	0.335	0.658	0.007	0.585	0.415	0
**Repetitive behaviour**	0.272	0.502	0.226	0	0.602	0.398	0.31	0.67	0.02	0.778	0.169	0.053
**Communica-tion difficulties**	0.257	0.532	0.211	0	0.435	0.565	0.255	0.679	0.066	0.591	0.377	0.032
**Safety concerns**	0.321	0.474	0.205	0	0.549	0.451	0.083	0.917	0	0.394	0.606	0
**Sensory issues**	0.235	0.523	0.242	0	0.451	0.549	0.083	0.904	0.013	0.615	0.385	0
**Education**	0.226	0.517	0.257	0.011	0.55	0.44	0.34	0.617	0.043	0.708	0.292	0

Class 1 (46.4% of the sample) was similar to the average pattern of response, with the probability for most domains to not change was approximately 50%, and to worsen or improve were roughly equivalent, approximately 25% chance each. Across all domains, there were probabilities of 37–64% of endorsing no change in functioning. Improvement on troubling behaviour showed the lowest probability of endorsement (15%) while the domains with the highest probability to improve was daily living (32%) and health problems (31%). The pattern was less variable for worsening, with the lowest probability to worsen was on troubling behaviour (22%) and the highest for daily living (41%) and sleep (32%). This class was labeled ‘Stable profile’.

In Class 2 (10.2% of the sample) there was an almost zero probability of worsening on any domain, and equal probability of endorsing ‘no change’ or ‘improvement’ in functioning on average across all domains. Improvement showed the highest probability of being endorsed on daily living skills (71%), diet/eating difficulties (73%), communication (57%), and sensory issues (55%). On the remaining domains, there was a 55–60% chance of being endorsed as no change. This class was labelled ‘Resilient profile’.

Classes 3 and 4 both showed nearly zero probability of improvement on any domain of functioning. Class 3 (26.8% of the sample) represented a high probability of endorsing ‘no change’ across all domains, particularly for safety concerns (92%), sensory issues (90%), health problems (85%), daily living (79%), troubling behaviour (78%), and diet/eating difficulties (74%). Worsened was endorsed with an average of 25% across all domains, with the highest probability to worsen on the following domains: sleep (41%), education (34%), mental health (34%) and social interaction difficulties (34%). This class was labelled ‘Somewhat worsening profile’.

Class 4 (16.4% of the sample) represented a high probability of endorsement of ‘worsened’ across all domains (46%-84%) apart from health problems and safety concerns where no change had a high probability of response (77% and 61%, respectively). The domains with the highest probability of being endorsed as worsening were mental health (84%), troubling behaviour (81%), repetitive behaviour (78%), and sleep (75%). The class was labelled ‘Clear worsening profile’.

### Multinominal logistic regression to examine association between candidate predictors and class membership

We performed a multinominal logistic regression to examine the association between candidate predictors and profile membership. Because classes 3 and 4 both reflected an absence of improvement, we combined these two classes as the reference category ‘Worsening profile’ to examine predictors of the two classes which reflected improved functioning. Table 4 details the results of the regressions examining the extent to which parenting self-efficacy, type of diagnoses, and ease in accessing schooling was associated with the likelihood of being classified into each of the participant profile (reference: combined Classes 3 & 4 i.e. ‘Worsening profile’), after controlling for two-parent status and income level.

**Table 4:** Multinominal logistic regression to examine association between candidate predictors and class membership.

Candidate predictors	Class 2 “Resilient profile” (n = 90) vs Combined Classes 3 & 4 “Worsening profile” (n = 381) OR (90% CI)	Class 1 “Stable profile” (n = 410) vs Combined Classes 3 & 4 “Worsening profile” (n = 381) OR (90% CI)
**Sociodemographic variables**	** **	** **
Two-parent household	2.35* **(1.02–5.41)**	0.73 (.50–1.07)
Annual household income ≥ $40k	1.97 (.89–4.33)	3.79*** **(2.31–6.20)**
**Hypothesis testing variables**	** **	** **
Diagnosis (reference: ASD/ID only)		
Diagnoses other than ASD/ID (n = 383)	2.13* **(1.08–4.19)**	2.86*** **(1.91–4.33)**
ASD/ID plus other diagnoses (n = 322)	0.85 (.55–2.05)	0.54*** **(.36 - .81)**
Confidence in helping child cope	1.65*** **(1.34–2.02)**	1.37*** **(1.20–1.60)**
Difficulty in accessing school (reference: neither easy nor difficult)		
Somewhat easy or very easy (n = 239)	3.18** **(1.62–6.22)**	1.71* **(1.12–2.62)**
Somewhat or very difficult (n = 411)	0.77 (.40–1.46)	0.51*** **(.36 –.74)**

***p < .001

**p < .01

*p < .05.

Two-parent status and household income were included as potential confounding variables and an initial model was run to test the associations with class membership. Children and youths in two-parent families were 2.4 times more likely to have the Resilient profile than the Worsening profile compared to those in single-parent families. Annual household income was not associated with Resilient versus Worsening profiles, but those reporting an annual income of more than or equal to $40k were 3.8 times more likely to have the Stable profile compared to the Worsening profile.

After controlling for two-parent status and income level, we found that type of diagnosis, parenting self-efficacy and ease in accessing schooling predicted membership in the Resilient compared to the Worsening profiles. Specifically, children and youths with non-neurodevelopmental conditions were 2.1 times more likely to have the Resilient than the Worsening profile compared to those with only ASD/ID. Children and youths whose caregivers reported higher parenting self-efficacy were 1.7 times more likely to have the Resilient than the Worsening profile. Those who had somewhat easy or very easy access to school were 3.2 times more likely to have the Resilient compared to the Worsening profile compared to those whose access to school was neither easy nor difficult.

Similarly, the predictors assessed were also associated with the Stable compared to the Worsening profile. Compared to children and youths with only ASD/ID, children and youths with non-neurodevelopmental conditions were 2.9 times more likely to be in the Stable than the Worsening profile, while children and youths with ASD/ID along with other conditions were twice more likely to belong in the Worsening than a Stable profile. Children and youths whose caregivers reported higher parenting self-efficacy were 1.4 times more likely to be in the Stable than the Worsening profile. Finally, compared to children and youths whose access to school was neither easy nor difficult, those who had somewhat easy or very easy access to school were 1.7 times more likely to be in the Stable compared to the Worsening profile, while those who had somewhat difficult or very difficult access to school were twice more likely to be in the Worsening than to a Stable profile.

## Discussion

We explored various profiles of changes in functioning among children and youth with disability across 12 domains during the COVID-19 pandemic based on caregiver report. Our findings highlighted a heterogeneous response to the COVID-19 pandemic. Approximately half of the caregivers in our sample reported a *Stable profile*, where the majority response was no change. Other caregivers (43% of the sample) reported a *Worsening profile*. We also identified a clear, albeit smaller subgroup (10%) with reported improvements in functioning during the pandemic, a pattern indicative of a *Resilient profile*.

This study presents the second largest sample size to date assessing a range of domains in this target population of children and youth with disability at a later timepoint in the COVID-19 pandemic (June-July 2020). The study is unique in assessing stability, worsening, and improving in a number of domains of functioning that have not been measured in this target population in previous studies. The reports of worsening in behaviour, nutrition, communication, sleep and mental health during the COVID-19 pandemic is consistent with existing evidence [4, 10, 11, 13, 48]. Other domains queried in this study were also reported to worsen in a proportion of participants, namely daily living skills, health problems, social interaction difficulties, repetitive behaviours/limited interests, safety concerns, sensory issues, and education. It is possible that a worsening in all these domains reflect an interlinking and cascading reaction to disruptions in established structure, routines, and services due to the pandemic [49]. Disrupted routines are associated with sleep problems [50], which in turn are linked with increased sensory issues, communication problems, issues in adaptive and challenging behaviours, and increased severity in autism symptoms in children with ASD [51]. Associations between reduced external structure in routines and increased repetitive and restrictive behaviours has been shown [52], potentially stemming from the need to regulate one’s sensory input from the environment, as evidenced by increased sensory hypersensitivities in individuals with more repetitive behaviours [53]. A worsening in social interaction difficulties and education may be linked with school/daycare closures during the pandemic [54]. Finally, reports of worsening of health problems and safety concerns may reflect concerns directly related to the COVID-19 disease and the SARS-CoV-2 virus. Interestingly, certain domains namely daily living skills and health problems, seemed to show a similar proportion of endorsement of worsening and of improvement. This underscores the variability in response to the pandemic, for some families the changes are associated with a worsening, but for others the changes may facilitate an improvement.

The largest profile identified in the present study we labeled *Stable* as the highest probability of response for each domain was no change. This profile most closely resembled the average response in the sample. This is consistent with studies which have found no change in group-level outcomes found in a longitudinal study [15]. This finding must be considered alongside the period of assessment in this study of June-July 2020, perhaps reflecting a relatively more stable period of adaptation compared to a more acute response early in the pandemic. A systematic review and meta-analysis of 65 longitudinal studies in mostly general populations showed that most mental health symptoms except for depression increased early in the pandemic, but declined over time to pre-pandemic levels by May-July 2020 [4]. It may be that an assessment closer to the onset of the pandemic would have shown greater change in symptoms. However, it is important to note that whilst individuals in this profile showed the highest probability of endorsing no change across the domains, they were also characterised by both worsening and improvement.

The combined *Worsening* profiles representing 43% of children and youths indicate that a substantial of individuals do not enter this period of stable adaptation but continue to worsen across multiple domains. The worsening domains were characterised by almost zero probability of showing improvement in any domain, and a very high probability of showing worsening in one of the classes. In particular, the domains with the highest probability to worsen in the *Clear Worsening* profile were mental health, troubling behaviour, repetitive behaviour, and sleep. Notably one longitudinal study found worsening sleep in autistic children and youths in May 2020 compared to pre-pandemic levels [29]. More sleep problems during the COVID-19 pandemic was associated with greater severity of autism symptoms pre-pandemic [11, 29] and was more likely reported in single-parent families [11]. As discussed further below, it is likely that these factors make it less likely for children and youths to adapt to the pandemic overall and thus also impact other domains beyond sleep found to worsen in this profile like mental health and troubling and repetitive behaviour.

Previous studies have reported on small subgroups of participants who improved in certain domains during the pandemic, potentially consistent with the small *Resilient* profile we found (10% of the sample). Within this Resilient profile, there was almost zero chance of worsening on any domain, above 70% chance of improving on diet/eating issues and daily living skills, and 55–60% chance of no change on the remaining domains. Approximately 10% of participants in Berard et al.’s study [11] reported improvements in nutrition. Insight from qualitative studies would suggest that the increase in quality family time afforded by some families during the pandemic allowed for a slower pace of life and the ability try new things [16, 55], such as making meals together [55].

In sum, using a data-driven approach across multiple domains of functioning, we found that children and youths with disability respond to the COVID-19 pandemic in heterogenous ways across multiple domains of functioning, including a pattern of resilience. Understanding the factors associated with this pattern of resilience would provide insight into which modifiable conditions would favour a more adaptive response to an adversity like the COVID-19 pandemic versus those that subject a child or youth to more vulnerability. After controlling for two-parent status and income level, we found that type of diagnosis, parenting self-efficacy and ease in accessing schooling were associated with membership of a child or youth in either the Resilient or Stable profiles compared to the Worsening profile.

Profiles of response to the COVID-19 pandemic differed depending on the child’s or youth’s specific diagnosis. When compared with children and youths with diagnoses *other than* ASD/ID, those with an ASD/ID diagnosis were more likely to belong to the Worsening rather than the Resilient profile. In addition, those with an ASD/ID diagnosis plus other diagnoses were also more likely to belong to the Worsening profile relative to the Stable profile compared to those with an ASD/ID diagnosis only. A possible explanation is that the disruptions in necessary services including schooling were more detrimental to children and youths with ASD/ID during the pandemic compared to those without ASD/ID, with the impact of disruptions amplified by the presence of other diagnoses in addition to ASD/ID. At the same time, stronger caregiver confidence in helping one’s child cope was associated with resilience in the child/youth. It is possible that *parenting* self-efficacy promotes resilience by improving the caregiver’s ability to access resources related to parenting that could improve/maintain the child’s or youth’s functioning. Interventions targeted to empower parents have shown improvements in child outcomes [56, 57], with an increase in positive parenting skills appearing to partially and significantly mediate the change in observed child problem behaviour [58]. In the “silver linings” study, parents reported a variety of coping strategies during the pandemic, namely applying routines and behavioural strategies, taking part in enjoyable family activities, practicing meditation, etc. [16]. These may all contribute to the role of parenting self-efficacy that we identified as potentially valuable for resilience in the child or youth.

Finally, as hypothesized, ease in access to schooling was associated with resilience, consistent with the literature showing that a powerful resource during an adversity is schooling [32]. The COVID-19 pandemic is unique in that access to schooling can be remote, which may or may not offer similar opportunities, and indeed may create additional stresses in a household, for example, in the need to supervise remote learning. Facilitating access to remote schooling requires equipping educational systems with tools to be able to better deliver remote schooling such as providing training to improve “digital competence” among teachers and students, implementing routines to promote closer partnership between teachers and caregivers, and sharing higher-quality and tailored resources along with more guidance on routines [59] for vulnerable students who may face additional barriers [60].

Consistent with other reports in the literature [35–39], the resilience profile was less likely for families with low-income and those in single-parent households. Low-income and single-parent families are likely facing a greater burden of childcare during the COVID-19 pandemic, considering they are less likely to have flexible work accommodations or be able to work remotely, thus more likely to have work disruptions and lost income to the pandemic, more likely to juggle childcare and financial responsibilities on their own, and are less likely to be supported in their child’s schooling, which all adds up to an additional burden to childcare with homeschooling [61–63]. Single parents in our sample were more likely to be women, known to shoulder childcare responsibilities. Even in two-parent households, a gender gap was likely widened by the COVID-19 pandemic [63–66].

Taken together, our findings identified variability in responses to adversity among children and youths with disability. By identifying potentially modifiable predictors of resilience among candidate predictors, we signal the potential for tailored supports for those with ASD/ID, through interventions that enhance caregiver empowerment, access to schooling, access to health and social services, and/or mitigate disparities resulting from social disadvantage.

### Limitations

While the strengths of the study are that we recruited a large sample of caregivers of children with disability recruited through various methods and located across Canada, collected within a narrow time frame, allowing a capture of a similar stage of exposure to the pandemic, the study had several limitations. Firstly, we recruited from a non-random, convenience sample. As a result, our sample may not be representative of the target population of children with disability in Canada. Another main limitation to the study is the reliance on participant-reported measures in a cross-sectional design. While caregivers are highly sensitive to changes in their child’s functioning, with their reported concerns meeting standards for developmental screening test specificity and sensitivity [67], we cannot rule out the possibility that the heightened stress levels during the COVID-19 pandemic can impact caregiver ratings of child functioning, as demonstrated in a previous study [68]. As such, the findings would be stronger if functioning was assessed from multiple sources. We also cannot rule out biases inherent to self-report measures and retrospective surveys such as recall bias [69]. Additionally, due to the cross-sectional design of the study we cannot infer causality between the candidate predictors and profiles of functioning, nor can we rule out the possibility of shared method variance to explain the association between the ratings of confidence in coping with the ratings of child functioning. Longitudinal studies measuring changes in child functioning from multiple sources measured at multiple timepoints during the pandemic would provide converging evidence of changes in child functioning related to the pandemic and direct evidence for resilience processes underlying observed trajectories of functioning. Future work to account for additional direct measures of adversity such as job loss, risk of infection, amount of financial support received, or other factors that could further clarify the response profile.

## Conclusions

We characterized four profiles of child functioning during the pandemic across multiple domains of functioning. Most respondents belonged to classes which reflected either stability in functioning, a moderate chance of worsening in certain domains, or high probability of worsening in most domains. However, we identified a small group who showed a high chance of improvement in certain domains and stability in other domains, which provides evidence for resilience in some children in the face of the COVID-19 pandemic. We found that resilience in children and youths with disability is associated with diagnostic type, parenting self-efficacy and support to schooling. The results highlight the need for services that promote self-efficacy in caregivers and support access to schooling to temper the negative impact of the pandemic to vulnerable populations, such as those with ASD or ID. The consistency of our findings with the broader disaster literature suggest that these variables are also likely ingredients in promoting resilience in children and youths in the face of future pandemics.
